# Treatment of uncomplicated malaria at public health facilities and medicine retailers in south-eastern Nigeria

**DOI:** 10.1186/1475-2875-10-155

**Published:** 2011-06-08

**Authors:** Lindsay J Mangham, Bonnie Cundill, Ogochukwu Ezeoke, Emmanuel Nwala, Benjamin SC Uzochukwu, Virginia Wiseman, Obinna Onwujekwe

**Affiliations:** 1 Department of Global Health and Development, London School of Hygiene and Tropical Medicine, London, UK; 2 Department of Infectious Disease Epidemiology, London School of Hygiene and Tropical Medicine, London, UK; 3 Health Policy Research Group, Department of Pharmacology and Therapeutics, College of Medicine, University of Nigeria (Enugu Campus), Enugu, Nigeria; 4 Department of Community Medicine, College of Medicine, University of Nigeria (Enugu Campus), Enugu, Nigeria; 5 Department of Health Administration and Management, College of Medicine, University of Nigeria (Enugu Campus), Enugu, Nigeria

## Abstract

**Background:**

At primary care facilities in Nigeria, national treatment guidelines state that malaria should be symptomatically diagnosed and treated with artemisinin-based combination therapy (ACT). Evidence from households and health care providers indicates that many patients do not receive the recommended treatment. This study sought to determine the extent of the problem by collecting data as patients and caregivers leave health facilities, and determine what influences the treatment received.

**Methods:**

A cross-sectional cluster survey of 2,039 respondents exiting public health centres, pharmacies and patent medicine dealers was undertaken in urban and rural settings in Enugu State, south-eastern Nigeria.

**Results:**

Although 79% of febrile patients received an anti-malarial, only 23% received an ACT. Many patients (38%) received sulphadoxine-pyrimethamine (SP). A further 13% of patients received an artemisinin-derivative as a monotherapy. An estimated 66% of ACT dispensed was in the correct dose. The odds of a patient receiving an ACT was highly associated with consumer demand (OR: 55.5, p < 0.001).

**Conclusion:**

Few febrile patients attending public health facilities, pharmacies and patent medicine dealers received an ACT, and the use of artemisinin-monotherapy and less effective anti-malarials is concerning. The results emphasize the importance of addressing both demand and supply-side influences on malaria treatment and the need for interventions that target consumer preferences as well as seek to improve health service provision.

## Background

Malaria remains a major cause of death and illness in children and adults in tropical settings. An integrated strategy is recommended which ensures access to treatment with effective anti-malarials, while also undertaking preventative measures that target vector control [1]. ACT became the recommended treatment for uncomplicated malaria, as resistance emerged to conventional monotherapies, including sulphadoxine-pyrimethamine (SP), chloroquine and amodiaquine, thereby reducing their therapeutic efficacy. Over the last decade, countries have revised their national malaria treatment policies to adopt ACT as the first-line recommended treatment for uncomplicated malaria. Although these policies are now well established, there are persistent problems with their implementation.

Evidence from several settings on malaria case management report problems with the choice of treatment, showing that ACT is often underused and many patients continue to receive less effective anti-malarials, such as SP [2-4]. There are also concerns about the availability and use of artemisinin monotherapy, as drug resistance is more likely to develop if artemisinin derivatives are taken without a partner drug [5,6]. Problems with the dispensing of malaria treatment have also been observed, with patients frequently receiving inadequate doses and without advice on how the medicines should be taken [2,7]. Ensuring accuracy of drug dispensing is particularly challenging for pharmacies and other drug retailers which typically stock a multitude of different types of ACT and the accurate dosage depends not only on the patient's age or weight but varies by brand depending on the formulation and composition of the active ingredients [8,9].

In Nigeria, it is estimated that children under five years of age have between two and four episodes of malaria each year, and ensuring prompt access to effective treatment is a key strategy of the Nigerian Federal Ministry of Health [10]. At the level of primary care, the national malaria treatment guidelines state that diagnosis should be based on symptoms using the Integrated Management of Childhood Illnesses (IMCI) classification [10]. Thus, patients presenting with febrile illness at health facilities without diagnostic testing available should be presumptively treated for malaria. ACT became the recommended treatment for uncomplicated malaria in 2005 and at this time new treatment guidelines and training materials were developed [10-12]. The first-line recommended treatment is artemether-lumefantrine (AL), though treatment with artesunate-amodiaquine (ASAQ), artesunate-mefloquine (ASMQ) and dihydroartemisinin-piperaquine (DHAPQ) are also considered acceptable [12]. The policy is also clear that SP is reserved for intermittent preventive treatment in pregnancy, and cases of severe malaria should be treated using quinine injection, artemether injection, or artesunate (either as an injection or suppository); otherwise the use of monotherapies is no longer recommended.

The provision and utilization of malaria treatment in south-eastern Nigeria is well researched, with evidence from household surveys, patient records and from health care providers at a range of health facilities showing that many febrile patients do not receive the recommended anti-malarial [4,13-16]. Much less is known about the quality of care provided at health facilities, and this study explores these concerns directly by collecting data from patients exiting public health facilities and medicine retailers. These types of facilities are the main providers of malaria treatment at the primary care level [14]. This paper describes the characteristics of patients and the health facilities they attend, and their experience of care, including the nature of the consultation, the provision of anti-malarial treatments and the quality of drug dispensing. The paper also investigates whether patient, health worker or facility factors are associated with receiving ACT.

## Methods

### Study area

The study was undertaken in two study sites in Enugu State in south-eastern Nigeria: Enugu urban (comprising of Enugu East, Enugu South and Enugu North local government areas (LGAs)) and Udi LGA. Enugu urban is the largest predominantly urban area in Enugu State, and Udi LGA is rural. Malaria is endemic in Enugu State, and occurs all year round. The people of Enugu are of Igbo ethnicity and speak the Igbo language. The activities of the majority of the population include farming, fishing, wine tapping, and poultry keeping and rearing of domestic animals; the main agriculture season runs from November to February.

ACT was introduced into the study site in 2005 by the State Malaria Control Programme. The implementation package consisted of training health workers on symptomatic diagnosis, change in antimalarial policy and rational prescription of antimalarials and was accompanied by a community awareness campaign.

### Study setting

The study was undertaken at public primary health facilities, private sector pharmacies and patent medicine dealers (PMDs) in Enugu State, south-eastern Nigeria [17]. The term PMD refers to retail outlets that are licensed to sell over the counter pharmaceutical products, though often hold a wider range of stock, and typically have no formal training [18]. Pharmacies and PMDs are a major source of malaria treatment [14,19]. These facilities are medicine retailers and do not routinely offer clinical care or diagnostic services. At the primary care level, presumptive treatment of malaria is recommended in febrile patients, as few public facilities offer malaria microscopy or RDTs. Primary health centres are usually staffed by community health officers and community health extension workers and supported by registered nurses and midwives [20].

### Study design

A stratified multistage cluster survey was conducted between July and December 2009. The survey sampling was clustered in 16 randomly selected communities and stratified by type of facility: i) public facilities including primary health centres, dispensaries and health posts, and ii) pharmacies and PMDs. Within each community all public primary health centres were included due to their small number. There are a large number of pharmacies and PMDs, and these were randomly selected with probability proportionate to size assuming that a total of 80 (out of 298) medicine retailers could be visited given the financial resources and time available. All health workers within each facility responsible for prescribing or dispensing medicines were included in the study.

A survey sample of 20 patients per public facility was calculated to estimate the primary outcome, the proportion of febrile patients receiving the recommended treatment for malaria, with a precision of +/- 13%, assuming that the variability (intra-cluster correlation, ICC) in treatment between facilities is 0.3 [21]. For pharmacies and PMDs, 14 patients per facility allows the primary outcome to be calculated with a precision of +/- 6.6% assuming the same degree of variation. The estimates assume a prevalence of 50% for the primary outcome and give the maximum range for precision (if the observed prevalence by higher or lower than 50% then greater precision would be achieved). The sampling was based on an enumeration of health facilities and their staff conducted in April 2009.

### Survey activities

In advance of the survey a field team visited each facility to explain the purpose of the survey to the head of the facility and obtain informed written consent. Informed consent was reconfirmed verbally on the day of the actual survey. The survey questionnaires were developed specifically for the study and pretested on a non-random sample of individuals with characteristics similar to those of the survey population but not chosen for inclusion in the survey. Survey teams were trained on procedures for conducting the survey and involved in the pretesting and revision of the questionnaires. Site supervisors monitored and supervised all aspects of data collection.

Data were collected using three structured approaches; a patient exit questionnaire, a health worker survey and a health facility audit. Written consent from patients and caregivers (who may or may not be accompanied by the patient) exiting the health facility was sought before screening to determine their eligibility to participate in the survey. An individual was considered eligible if s/he reported seeking treatment for a fever or if s/he had received an ACT. Treatment may be sought for themselves, a child or another person who is not present (the latter applies only at medicine retailers). Individuals that were exiting a health facility were assessed in turn until the patient quota was reached. All workers that were involved in prescribing or dispensing malaria treatment and were available at the time of the survey were invited to complete the health worker survey and written consent was obtained from all participants.

The patient exit questionnaire collected data on the patient's prior treatment seeking and use of anti-malarials, reasons for attendance, the consultation and diagnosis, prescriptions and medicines received, the cost of treatment seeking and the demographic characteristics of the patient. The health worker questionnaire captured data on their characteristics, access to in-service training and national malaria treatment guidelines, malaria knowledge and treatment practices. The health worker survey was conducted once all the patient exit questionnaires had been completed to ensure that the treatment received by patients was not influenced by the content of the health worker questionnaire and the patient exit data best reflects current prescribing practices. The health facility audit was conducted following the health worker survey and collected data on the characteristics of the health facility, diagnostic services, management and procurement of medicine, including the availability of ACT.

### Definitions

The treatment received by patients was assessed against the national malaria treatment guidelines, which recommends that patients with a fever are presumptively treated with an ACT, with the exception of pregnant women in the first trimester. The accuracy of the ACT dose provided to patients was assessed in accordance with dosage recommendations based on the patient's age and the type and composition of ACT received. Thus, the analysis takes into account that the correct number of tablets (or powder sachets) varies by brand, the amount of active ingredients contained in each tablet and whether they are co-formulated or co-blistered. Suspensions were excluded from the analysis on dosing. As patient age was used as a proxy for weight [11,22] this may cause some error in estimating the accuracy of dosing among children, though this would not apply to adults. Patient knowledge on the dose regimen was ascertained by asking the patient or their caregiver to explain how and when the medicine should be taken. Knowledge was considered accurate if they reported the number of tablets (or powder sachets) which should be taken per day over 3 days that corresponds to the specific brand of ACT received, and the patient's age. Suspensions were excluded from the analysis due to the difficulties in accessing the accuracy of the correct dose.

### Statistical analysis

Data were entered and verified using Microsoft Access 2007 (Microsoft Inc., Redmond, Washington) and analysed using STATA version 11.0 (STATA Corporation, College Station, Texas) that allows for complex survey design by identifying different probabilities of selection (sampling weights), clustering and stratification (applying the prefix svy) [23]. Thus, all percentages and odds ratios reported are population-average estimates which have been adjusted to take into account the stratification, clustering and sampling weights of the study design. The weights are equal to the inverse probability of being sampled and took into account the sampling probabilities at the facility, health worker and patient level. At the patient level, number of days it took to recruit patients was used to create a proxy for the volume of patients, with the less time indicative of a larger facility.

Treatment outcomes by strata were compared using the Rao and Scott chi-square correction [24]. Survey logistic regression was used to assess factors associated with receiving the recommended treatment. The following were investigated for their potential association: characteristics of the patient and health worker, patient consultation, and the resources available at the health facility (all factors are listed in Table six). Factors associated with receiving the recommended treatment were investigated in the multivariable model if the univariable association was statistically significant at the 10 percent level, or the odds ratio was less than 0.5 or greater than 1.5. Factors were retained in this multivariable model if they remained significantly associated at the 10% level of significance or with an adjusted odds ratio less than 0.5 or greater than 1.5. Models were compared using an adjusted Wald test. Pregnant women and children under the age of 6 months were excluded from the analysis because the national malaria treatment guidelines have alternative recommendations for these groups.

### Ethical approval

Ethical approval for this study was obtained from the ethics committees of University of Nigeria and London School of Hygiene and Tropical Medicine.

## Results

### Patient characteristics

**Figure 1:**
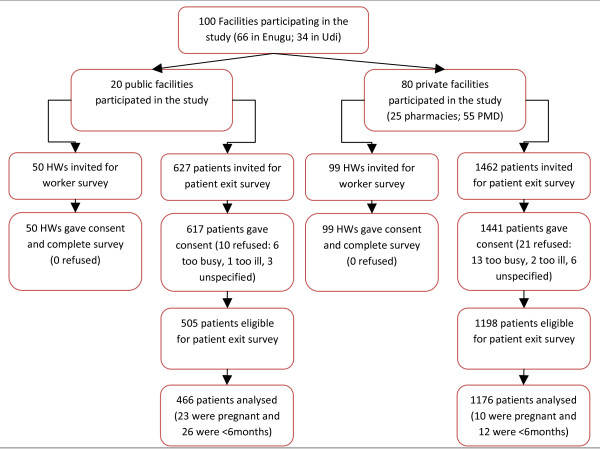
**Study population**.

Data were collected from 100 health facilities and the analysis is based on exit data collected from 1,642 febrile patients attending public facilities and medicine retailers and 149 health workers (Figure 1). There was notable variation in the characteristics of patients attending the different types of health facility (Table 1). More than half (57%) of the patients treated at public health facilities were children, while 80% of the cases presenting at pharmacies and PMDs were adults. Treatment-seeking also varied by education levels and socioeconomic status (SES), with respondents surveyed at medicine retailers more likely to have tertiary education and be of a higher wealth quintile. At medicine retailers81% of patients reported it was the first time that they had sought treatment for this illness episode, and 43% had sought treatment on the same or day following the onset of symptoms. While at public facilities 61% of patients at public facilities were seeking treatment for the first time and the time before treatment was much longer, with only 16% seeking treatment on the same or day following the onset of symptoms. When asked about their choice of health facility, many respondents said that they had sought treatment at this facility for past illnesses (54%) and it was convenient (55%). In addition, patients at public health facilities often mentioned the lower cost of treatment, while the reputation of the provider and the availability of drugs were more often cited at medicine retailers.

**Table 1:** Patient Characteristics by type of facility*

	Public		Medicine retailer		Total		
	
	N = 466	%	N = 1176	%	N = 1642	%	P value
**Patient gender^i^**							

Male	218	45.4	624	56.4	842	55.5	0.006

Female	242	54.6	536	43.6	778	44.5	

**Patient age**							

>15 years (adult)	185	42.8	913	79.6	1098	76.7	<0.001

10-15 years	27	6.6	85	7.3	112	7.3	

5-9 years	61	12.6	71	4.6	132	5.3	

<5 years	193	38.0	107	8.4	300	10.7	

**Patient socioeconomic status^ii^**							

Poorest quintile	223	46.4	343	15.7	566	18.1	<0.001

Second quintile	97	20.4	219	20.0	316	20.1	

Third quintile	55	11.8	219	21.3	274	20.6	

Fourth quintile	56	13.5	189	21.2	245	20.6	

Richest quintile	35	8.0	206	21.8	241	20.7	

**Education level of patient (or caregiver)^iii^**							

No formal education	25	5.8	41	1.3	66	1.6	<0.001

Primary education	116	28.0	198	12.5	314	13.7	

Secondary education	213	46.1	469	39.7	682	40.2	

Tertiary education	93	20.0	445	46.6	538	44.5	

**Was first time sought treatment^iv^**							

Yes	269	61.4	896	81.7	1165	81.1	<0.001

No	196	38.7	273	18.3	469	19.9	

**Number of days since start of symptoms^v^**							

None (same day)	14	3.3	234	22.1	248	20.6	<0.001

1 day	71	12.6	259	21.2	330	20.5	

2 days	93	19.3	228	21.2	321	21.1	

3-5 days	202	45.4	284	23.5	486	25.3	

6+ days	85	19.4	169	12.0	254	12.6	

**Reasons given for choice of health facility^vi^**							

Convenient	229	49.2	617	55.9	846	55.4	0.284

Used previously	243	48.6	732	54.7	975	54.2	0.346

Good reputation	145	30.1	529	49.6	674	48.1	0.002

Availability of drugs	127	23.3	548	48.7	675	46.7	0.001

Inexpensive	206	46.2	178	13.3	384	15.9	<0.001

Qualification of staff	132	24.7	174	12.9	306	13.9	0.011

i missing 22 responses: 6 from public and 16 from medicine retailer

ii Principal components analysis was undertaken to generate a SES index based on household asset ownership [33]. The SES index was disaggregated into wealth quintiles.

iii missing 42 responses: 19 from public and 23 from medicine retailer

iv missing 8 responses: 1 from public and 7 from medicine retailer

v missing 3 responses: 1 from public and 2 from medicine retailer

vi more than one reason could be given

### Health facility and health worker characteristics

The provision of basic equipment, such as weighing scales and thermometers was good in public health facilities, though more mixed in pharmacies and PMDs (Table 2). Very few health facilities offered malaria microscopy testing and none of the health facilities surveyed used RDTs. At the time of the survey, all health facilities reported that they had anti-malarials in stock, and field staff verified that ACT was in stock in 80% of health facilities. There was some variation by facility type, with 71% of public health centres and 89% of pharmacies and PMDs stocking at least one ACT. Two-thirds of health facilities had artemether-lumefantrine (AL) available, though other types of ACT were common in medicine retailers. Artemisinin monotherapy was available in 96% of medicine retailers while the vast majority (90%) of facilities also had SP as well as other types of anti-malarials available; these included less effective conventional treatments such as chloroquine and amodiaquine.

**Table 2:** Facility Characteristics

	Public		Medicine retailer		Total		
**HEALTH FACILITIES**	**N = 20**	**%**	**N = 80**	**%**	**N = 100**	**%**	**P value**

**Equipment and services available**							

Weighing scale†	19	94.1	36	50.0	55	53.1	<0.001

Thermometer	19	94.2	35	46.5	54	49.8	<0.001

Microscopy services†	3	12.8	0	-	3	0.9	<0.001

RDT	0	-	0	-	0	-	-

**Availability of anti-malarials**							

Any anti-malarial	20	100.0	80	100.0	100	100.0	-

Artesunate monotherapy	5	24.6	76	96.1	81	91.1	<0.001

Sulphadoxine Pyrimethamine (SP) †	18	89.8	71	89.9	89	89.9	0.982

Chloroquine	14	71.1	77	96.8	91	95.0	<0.001

Quinine	4	20.3	70	89.1	74	84.3	<0.001

Amodiaquine	3	14.4	60	85.3	63	80.3	<0.001

Any type of ACT	14	71.1	66	89.6	80	88.3	<0.001

Artemether Lumefantrine (AL)	12	65.6	64	79.6	66	78.4	<0.001

Artesunate Amodiaquine (ASAQ)	2	8.6	57	78.8	59	73.9	<0.001

Artesunate Mefloquine (ASMQ)	0	-	35	52.9	35	49.2	<0.001

Artesunate Sulphadoxine-Pyrimethamine (ASSP)	0	-	18	28.4	18	26.4	<0.001

Dihydroartemisinin-Piperaquine (DHAPQ)	0	-	55	78.0	55	72.6	<0.001

**Median cost of ACT **(& IQ range)							

Adult dose of any ACT	-		600 (350, 750)		600 (350, 750)		-

Child dose of any ACT	-		350 (260, 600)		350 (250, 600)		-

Adult dose of AL	-		750 (650, 835)		750 (650, 820)		-

Child dose of AL	-		650 (580, 750)		650 (520, 700)		-

**HEALTH WORKERS**	N = 50	%	N = 99	%	N = 149	%	P value

Doctor	7	14.0	0		9	1.7	<0.001

Nurse or Midwife	7	14.0	7	7.3	14	8.1	

Community Health Officer	14	28.0	1	1.3	15	4.5	

Community Health Extension Worker	22	44.0	3	4.1	25	8.9	

Pharmacist‡	-		3	3.9	3	3.4	

PMD or pharmacy attendant‡	-		85	83.4	85	73.5	

HW has attended malaria training in past 3 years	13	24.6	31	33.0	44	31.9	0.011

HW has access to malaria treatment guidelines	15	30.9	4	5.2	19	8.5	<0.001

HW accurately reported ACTs are the recommended treatment for uncomplicated malaria	38	77.2	44	62.2	82	65.4	<0.001

† missing response from one pharmacy

‡ not applicable in public facilities

All the public facilities reported that ACT was available to patients free of charge. In pharmacies and PMDs the median price of an ACT was 600 Naira for an adult dose and 350 Naira for a child dose (which is approximately equivalent to USD $4.00 and USD $2.30). The median price of AL was higher at 750 Naira for an adult dose and 650 Naira for a child dose (equivalent to USD $6.00 and USD $4.30).

Just under half (44%) of workers in public facilities were community health extension workers, semi-skilled health workers trained in primary care, and while junior to the other cadres listed in Table 2 may prescribe treatment or undertake minor procedures [20]. In the medicine retailers the majority (83%) described themselves as patent medicine dealers or pharmacy attendants.

Knowledge of malaria treatment was variable, though better in public facilities, as 80% of workers in public facilities reported that ACT is the recommended treatment for uncomplicated malaria, compared to 62% of workers in pharmacies and PMDs. Moreover, less than one in three health workers surveyed had attended an in-service malaria training workshop over the past three years and relatively few (9%) had access to the malaria treatment guidelines (31% of public health workers and 5% of health workers at pharmacies and PMDs).

### Patient consultation, prescription and requests for medicine

The nature of the patient's consultation differed by type of health facility (Table 3). In public health facilities 95% of respondents reported the health workers were told of the patient's symptoms, and 90% reported that they had told the health worker about the patient's fever. Patients reported to have been physically examined in 65% of cases, 50% had their temperature taken, though just 6% of patients were tested for malaria. At public facilities with microscopy testing available 21% of patients were tested for malaria. In pharmacies and PMDs patients were rarely examined (6%) or tested (<1%), though in 32% of cases health workers were told about the patient's symptoms and asked further questions.

**Table 3:** Patient consultation

	Public	Medicine retailer	Total	
	
	N = 466	N = 1176	N = 1642	
	
	% (95% CI)	% (95% CI)	% (95% CI)	P value
**Patient reported consultation**				

Told HW about patient symptoms	94.9 (94.4-95.4)	44.3 (36.9-52.0)	48.3 (41.3-55.4)	<0.001

Told HW that had a fever	89.8 (87.2-91.9)	40.3 (33.0-48.2)	44.3 (37.1-51.4)	<0.001

HW asked follow up questions about patient's symptoms	79.9 (76.3-83.0)	32.0 (25.0-39.9)	35.7 (29.1-43.1)	<0.001

Patient was physically examined	65.1 (48.8-78.5)	6.2 (3.5-10.5)	10.8 (7.8-14.5)	<0.001

Patient had temperature taken	49.7 (38.6-60.9)	1.7 (0.6-4.9)	5.5 (3.9-7.8)	<0.001

Patient tested for malaria at this facility	5.8 (3.6-9.3)	0.2 (0.0-1.4)	0.7 (0.3-1.3)	<0.001

**Patient requests for medicine**				

% of patients that asked for:				

any type of medicine	2.8 (1.9-4.2)	65.4 (57.9-72.3)	60.5 (53.7-66.9)	<0.001

an anti-malarial	1.2 (0.7-2.1)	58.3 (51.1-65.2)	53.8 (47.3-60.2)	<0.001

any ACT	0.9 (0.4-1.9)	16.0 (11.1-22.5)	14.8 (10.3-20.9)	<0.001

Artemisinin monotherapy	0	11.5 (8.6-15.3)	10.6 (8.0-14.1)	0.162

Amodiaquine	0	1.6 (0.8-3.4)	1.5 (0.7-3.2)	0.402

Chloroquine	0.1 (0.0-0.4)	1.3 (0.6-2.7)	1.2 (0.6-2.5)	<0.001

Quinine	0	0.7 (0.2-2.6)	0.7 (0.2-2.4)	0.631

SP	0.1 (0.1-0.1)	26.2 (21.5-31.5)	24.1 (19.8-29.1)	<0.001

**Anti-malarial prescriptions**				

% patients prescription (from any facility)	94.2 (92.4-95.6)	15.4 (10.7-21.5)	21.6 (17.1-26.9)	<0.001

% patients that received prescription from this facility	94.2 (92.4-95.6)	1.8 (0.7-4.0)	8.8 (7.3-11.0)	<0.001

% patients that were prescribed*:		-	-	-

an anti-malarial^†^	78.4 (72.6-83.3)	-	-	-

any ACT^‡^	34.0 (21.9-48.7)	-	-	-

Artemisinin monotherapy	4.7 (3.1-7.1)	-	-	-

Amodiaquine	1.1 (0.9-1.5)	-	-	-

Chloroquine	3.3 (0.8-11.6)	-	-	-

Quinine	0.2 (0.0-3.1)	-	-	-

SP	34.7 (21.7-50.6)	-	-	-

* Reported only for public health facilities

† At public facilities 73.4% of children under five years were prescribed an antimalarial.

‡ At public facilities 51.5% of children under five years were prescribed an ACT. At public health facilities with ACTs in stock 43.8% of patients (all ages) and 57.9% of children under five years were prescribed an ACT.

The majority of patients attending public facilities had medicines prescribed and in 78% of cases the prescription was for an anti-malarial. ACT was prescribed to 34% of patients seeking treatment, though as many patients were prescribed SP, which is no longer recommended for treating malaria.

At pharmacies and PMDs, 15% of patients had a prescription and patients often asked for a specific medicine. At these facilities, 58% of patients attending asked for an anti-malarial. Patients often asked for SP (26%), though also requested ACT (16%) and artemisinin-monotherapy (12%). Almost all (96%) of those patients that asked for an anti-malarial also received the medicine they had requested.

### Malaria treatment received by patients

Overall, the majority of patients received an anti-malarial, though ACT was received by only 22% of all patients attending health facilities and by 29% of children under five years of age (Table 4). SP is no longer recommended, though still frequently used, and 38% of patients had received this medicine. At public facilities, differences were observed between the proportion of patients that were prescribed and received antimalarials at facilities which had ACT in stock. The proportion of patients that received an antimalarial at public facilities was also low compared to the medicine retailers. There were, however, few differences between the proportions of patients receiving ACT and SP at public health facilities and medicine retailers, though patients were more likely to receive oral artemisinin monotherapy at medicine retailers than public facilities (14% compared to 2%, p < 0.001). Other anti-malarials, such as chloroquine, amodiaquine and quinine were rarely received by patients. By type of ACT, AL (44%) was most often dispensed and was widely used in the public sector. In medicine retailers, AL was regularly dispensed, though patients also received ASAQ and DHAPQ.

**Table 4:** Anti-malarials received

	Public	Medicine retailer	Total	
	
	% (95% CI)	% (95% CI)	% (95% CI)	P value
				

**Anti-malarials received (all ages)**	N = 466	N = 1176	N = 1642	

% of patients (of all ages) that received:				

an anti-malarial	54.2 (44.1-63.9)	81.5 (76.2-85.8)	79.3 (74.5-83.4)	<0.001

any ACT†	17.3 (9.0-30.5)	22.8 (17.2-29.7)	22.4 (17.0-28.8)	0.378

Artemisinin monotherapy*	2.0 (0.8-5.2)	14.4 (11.4-18.0)	13.4 (10.6-16.7)	<0.001

Amodiaquine	0.1 (0.00-1.0)	2.0 (0.9-4.6)	1.9 (0.8-4.2)	0.002

Chloroquine	2.4 (0.5-10.6)	3.3 (2.0-5.4)	3.2 (2.0-5.2)	0.673

Quinine	0	0.9 (0.3-2.2)	0.8 (0.3-2.0)	0.501

SP	33.6 (20.9-49.1)	38.2 (31.8-45.1)	37.9 (31.8-44.3)	0.546

				

**Anti-malarials received (children < 5 yrs only)**	N = 193	N = 107	N = 300	

% of children <5 years that received:				

an anti-malarial	33.2 (20.0-49.7)	80.2 (63.6-90.4)	67.1 (53.7-78.1)	0.001

any ACT‡	21.3 (9.9-39.9)	31.6 (18.2-49.0)	28.7 (18.0-42.6)	0.329

Artemisinin monotherapy*	2.0 (0.2-14.5)	10.2 (3.4-26.8)	7.9 (2.9-20.2)	0.110

Amodiaquine	0.2 (0.0-3.0)	9.3 (2.3-30.6)	6.7 (1.6-23.8)	0.001

Chloroquine	1.5 (0.7-3.2)	12.6 (4.2-32.1)	9.5 (3.5-23.5)	0.001

Quinine	0	4.5 (0.6-26.8)	3.23 (0.5-20.2)	0.497

SP	9.2 (3.7-21.0)	19.9 (8.5-40.0)	16.9 (8.1-32.0)	0.176

				

**Type of ACT received**	N = 105	N = 210	N = 315	

% AL	96.5 (92.5-98.4)	40.2 (26.9-55.1)	43.6 (31.0-57.2)	<0.001

% ASAQ	3.0 (1.4-6.2)	28.5 (16.1-45.2)	26.9 (15.3-42.8)	<0.001

% DHAPQ	0	24.3 (15.1-36.5)	22.8 (14.3-34.3)	0.074

% ASMQ	0.5 (0.1-1.9)	4.1 (0.9-16.6)	3.9 (0.9-15.5)	0.019

% ASSP	0	2.9 (0.7-11.5)	2.8 (0.7-10.8)	0.648

† At facilities with ACTs in stock 27.0% (20.4-34.7%) of patients at public facilities and 24.2% (17.9-31.9%) of patients at medicine retailers received an ACT

‡ At facilities with ACTs in stock 31.7% (22.0-43.4%) of children under five years at public facilities and 32.5% (18.0-51.3%) of children under five years at medicine retailers received an ACT

* This was in tablet form at medicine retailers.

### Quality of dispensing of ACT

Two-thirds (66%) of all types of ACT dispensed were estimated to be in the correct dose, while 58% of ACT dispensed were in the correct dose and the patient (or their caregiver) accurately reported how the medicine should be taken (Table 5). Given the challenges in estimating the accuracy of ACT dosage in children, the results are also presented for febrile adults receiving ACT. Overall the results are reasonably similar, with 56% of ACT received in the correct dose and by patients that had accurate knowledge of how to take the medicine. Very few patients receiving an ACT were told of any side effects associated with the medicine.

**Table 5:** Quality of dispensing for patients that received an ACT

	Public	Medicine retailer	Total	
	
	% (95% CI)	% (95% CI)	% (95% CI)	P value
				

All febrile patients that received an ACT*	N = 100	N = 176	N = 276	

% accurate dose^†^	75.8 (70.6-80.2)	65.5 (50.1-78.1)	66.2 (51.8-78.0)	0.135

% patient has accurate knowledge of treatment regimen^‡ i^	68.3 (63.7-72.6)	58.5 (41.6-73.6)	59.2 (43.4-73.3)	0.218

% patients with accurate dose and knowledge of treatment regimen^ii^	66.8 (61.3-71.9)	57.2 (40.3-72.5)	57.8 (41.7-72.4)	0.236

% patients that reported were told of side effects^iii^	1.5 (1.2-1.9)	3.0 (0.6-14.0)	3.0 (0.6-12.9)	0.357

				

Febrile adults that received an ACT*	N = 21	N = 125	N = 146	

% in accurate dose^†^	72.8 (68.6-76.6)	62.0 (43.5-77.5)	62.2 (44.2-77.4)	0.165

% patient has accurate knowledge of treatment regimen^‡ iv^	72.8 (68.6-76.6)	55.4 (36.3-73.0)	55.8 (37.1-73.0)	0.051

% patients with accurate dose and knowledge of treatment regimen^iv^	72.8 (68.6-76.6)	55.4 (36.3-73.0)	55.8 (37.1-73.0)	0.051

% patients that reported were told of side effects^v^	6.2 (5.0-7.7)	4.2 (0.8-18.5)	4.2 (0.9-17.8)	0.570

* excludes suspensions and syrups and limited to cases for which have data on dosage

† defined as dose that is consistent guidance on dosage by patient age.

‡ defined as patient reports treatment regimen is consistent with guidance on dosage by patient age

i missing 10 observations (2 from public and 8 from medicine retailers)

ii missing 12 observations (4 from public and 8 from medicine retailers)

iii missing 6 observations (2 from public and 4 from medicine retailers)

iv missing 2 observations (from medicine retailers)

v missing 3 observations (from medicine retailers)

### Factors influencing treatment received by patients

The odds of a febrile patient receiving an ACT were significantly associated with whether the patient had a prescription, asked for an ACT, the patient's gender, and the education level of the patient (or their caregiver) (Table 6). Patients were also significantly more likely to receive an ACT at health facilities that were better equipped, and had one or more health workers that knew ACT was recommended for uncomplicated malaria. Patients that chose the health facility because it was convenient or relatively inexpensive were significantly less likely to receive an ACT. Of all the variables considered in the univariable analysis, patients asking for ACT had by far the highest odds ratio of 53.3 (15.9-179.1, p < 0.001). This variable remained highly significant in the multivariable model with an odds ratio of 55.5 (15.0-205.6, p < 0.001), though the other significant variables were the patient's gender, the education level of the patient (or caregiver), whether the facility had a thermometer available, and whether the facility had health workers that knew ACT was recommended.

**Table 6:** Factors influencing whether a patient received an ACT

Variable				Univariable Analysis			Multivariable Analysis		
		n/N	%	OR	95% CI	P value	OR	95% CI	P value
**Study Site**	Enugu	211/989	22.7	1.71	(0.81-3.61)	0.148			
	Udi	71/531	14.7	1.0					

**Patient characteristics**									

Gender	Male	173/795	25.3	1.63	(1.00-2.65)	0.051	1.91	(1.02-3.55)	0.045
	Female	109/725	17.2	1.0			1.0		

Age Group	>15 yrs	157/1023	19.8	1.0		0.336			
	10-15 yrs	16/104	20.7	1.06	(0.44-2.53)				
	5-9 yrs	38/124	37.8	2.46	(0.90-6.74)				
	<5 yrs	71/269	29.0	1.65	(0.88-3.09)				

Quintile	Richest	57/230	28.8	2.35	(1.12-4.96)	0.201			
	Fourth	48/217	26.2	2.07	(0.99-4.35)				
	Third	48/257	18.7	1.34	(0.58-3.12)				
	Second	61/297	19.8	1.44	(0.64-3.25)				
	Poorest	68/519	14.6	1.0					

Education Level	No formal	3/61	2.1	0.09	(0.02-0.31)	0.001	0.13	(0.03-0.50)	0.045
	Primary	45/297	19.9	1.0			1.0		
	Secondary	113/651	19.1	0.95	(0.47-1.95)		0.81	(0.35-1.85)	
	Tertiary	121/511	25.4	1.37	(0.70-2.70)		0.84	(0.41-1.73)	

First-time go for treatment	Yes	207/1091	21.5	0.93	(0.50-1.73)	0.811			
	No	75/429	22.7	1.0					

Time before treatment	Same day	30/229	15.5	1.0		0.368			
	1 day	58/302	17.8	1.17	(0.49-2.83)				
	2 days	60/307	23.0	1.62	(0.78-3.38)				
	3-5 days	95/446	28.0	2.11	(0.97-4.58)				
	6+ days	39/236	23.4	1.66	(0.64-4.29)				

**Consultation with health worker (HW)**									

HW told of symptoms	Yes	170/1030	18.0	0.65	(0.34-1.22)	0.162			
	No	112/490	25.4	1.0					

HW is told of patient's fever	Yes	165/961	19.2	0.76	(0.41-1.43)	0.373			
	No	117/559	23.8	1.0					

HW asks follow up Qs	Yes	139/700	23.6	1.33	(0.68-2.61)	0.376			
	No	143/819	18.5	1.0					

Patient is examined	Yes	83/370	19.5	0.88	(0.42-1.87)	0.724			
	No	199/1150	22.0	1.0					

Takes patient temperature	Yes	53/251	19.1	0.90	(0.44-1.85)	0.755			
	No	229/1269	21.9	1.0					

Patient has a prescription	Yes	99/327	42.1	3.51	(1.77-6.95)	0.001			
	No	183/1193	17.1	1.0					

Asked for ACT	Yes	114/138	86.2	53.28	(15.9-179.1)	<0.001	55.47	(15.0-205.6)	<0.001
	No	168/1382	10.6	1.0			1.0		

**Health facility characteristics**									

Type of facility	Public	94/430	16.6	0.70	(0.29-1.67)	0.385			
	Retailer	188/1090	22.2	1.0					

Weighing scale available	Yes	216/892	27.2	2.13	(1.05-4.32)	0.037			
	No	66/628	14.6	1.0					

Thermometer available	Yes	198/872	27.5	1.94	(1.01-3.71)	0.046	1.99	(0.94-4.18)	0.068
	No	84/648	16.2	1.0			1.0		

Offer malaria microscopy	Yes	17/70	24.0	1.12	(0.77-1.63)	0.519			
	No	265/1430	21.7	1.0					

**Facility has one or more HWs that...**									

... have attended malaria training	Yes	137/660	22.0	1.03	(0.52-2.02)	0.927			
	No	145/860	21.6	1.0					

... have access to guidelines	Yes	66/236	19.2	0.83	(0.51-1.34)	0.413			
	No	216/1284	22.0	1.0					

... know ACT is recommended	Yes	251/1136	24.8	2.40	(1.03-5.57)	0.043	2.47	(0.91-6.73)	0.073
	No	31/384	11.5	1.0					

## Discussion

There is great need to improve the quality of care for uncomplicated malaria in south-eastern Nigeria. Parasitological diagnosis was available in only 3% of facilities and while the national malaria treatment guidelines recommend presumptive treatment of a fever with ACT when malaria tests are not available, less than a quarter (22%) of febrile patients attending facilities received the recommended treatment. Moreover, the estimates show that only 58% of patients that received ACT were given the correct dose and knew how the medicine should be taken. Inadequate dosing and poor compliance to treatment regimens will reduce the efficacy of the treatment taken and may contribute to the development of drug resistance [25].

After four years with ACT as the recommended first-line antimalarial, these results at public health facilities are extremely concerning. In Kenya and Zambia poor quality treatment practices were observed at public and mission facilities soon after ACT was introduced as first-line, though subsequent studies up to five years later show improvements in the proportion of patients that are prescribed and receive ACT [3,7,26]. As found elsewhere, the proportion of patients that were prescribed or received an ACT seems low given the availability of ACT at health facilities and the proportion of health workers that knew ACT was recommended [3,7,27,28]. It was also interesting to note that only half of patients at public facilities that were prescribed an ACT also received one, though it is not clear why this occurred: 34% of patients at public facilities were prescribed an ACT, while 17% received an ACT. The discrepancy is only partially explained by the availability of ACT and is unlikely to reflect the cost of treatment, as ACT is provided to patients in public facilities without charge.

There were some problems with the availability of ACT: 70% of public facilities and 83% of pharmacies and PMDs had at least one ACT in stock at the time of the survey. While the availability of ACT in the public sector was not as high as has been reported in Angola, Kenya, or Uganda [7,9,28-30], the availability of ACT in the study sites was much higher than the Nigerian national average from 2008, when it was found that 38% of public health facilities had ACT in stock. The availability of ACT at private sector outlets was found to be higher than the Nigerian national average from 2008, which reported 78% of pharmacies and 19% of PMDs had ACT in stock [9]. It is concerning to find that artemisinin monotherapy is widely available in medicine retailers and that many patients request this medicine. The use of oral artemisinin monotherapy in 13.4% of patients is also a major concern, since its use without a combination therapy can lead to the development of drug resistance [5,6].

Differences observed between the characteristics of patients by type of health facility are broadly consistent with evidence from household surveys conducted in Nigeria on malaria treatment seeking. For example, rural-urban differences, the education of caregivers, and socioeconomic status have been found to be important determinants of where treatment is sought [14,15,31-33]. Other studies have also shown that urban residents were more likely to obtain ACT [15] and individuals of higher levels of education and socioeconomic status were more likely to have correct knowledge of malaria treatment [31].

Differences between the facility types in the resources available and the patient's consultation were much as expected, with patients attending public health facilities more likely to discuss symptoms and be examined. Similarly, as pharmacies and PMDs are retail outlets it is not surprising that many lacked weighing scales and thermometers and that patients often asked for specific medicines. Moreover, it was expected that health workers in public facilities would have better access to the malaria treatment guidelines and be more likely to know that ACT is recommended for uncomplicated malaria.

The odds of a febrile patient receiving an ACT were positively associated with the health workers knowledge of the treatment guidelines, though there is no evidence of an association between access to treatment guidelines and attendance at malaria training. It should be noted that these variables were defined at the facility-level because in many cases it was not possible to link patients to the health worker that prescribed or recommended treatment, either because the health worker was absent at the time of the survey or because several health workers attended to the patient.

The treatment received by patients from medicine retailers was often driven by consumer requests for a specific medicine, and the odds of a febrile patient receiving an ACT were extremely high if the patient or their caregiver had asked for one. Previous studies from Nigeria have also highlighted the importance of patient demand. For example, Onwujekwe *et al *reports that 40% of providers across a range of primary health facilities said requests by patients influenced the type of drug provided [4]. Qualitative research with patent medicine dealers undertaken by Okeke *et al *also highlighted that patients often ask for specific medicines and the doses of anti-malarial drugs can be determined by patient's ability to pay [18]. Patients' requests for specific medicine at medicine retailers were likely to include cases for which treatment had been prescribed elsewhere, though as only 15% of patients had a prescription other factors are likely to be relevant and there would be merit in further examining the role of patient demand in influencing the choice of treatment for uncomplicated malaria in private sector facilities.

## Conclusions

ACT became the recommended treatment for uncomplicated malaria in 2005, though they remain underused, and less than a quarter of febrile patients attending health facilities in this study received ACT. Although there is increasing emphasis on the parasitological rather than symptomatic diagnosis of malaria, the study suggests that there is a need for interventions that also focus on choice of treatment to ensure that patients with malaria receive the recommended anti-malarial, irrespective of the diagnostic method. Improving the provision of health services should also address the quality of dispensing, and ensure that health workers can accurately determine the correct dose across a range of different brands and types of ACT. Concurrently attention needs to be given to the high availability and use of artemisinin monotherapy, as well as the continued use of less effective treatments, particularly SP. Consideration should also be given to the role of patient demand in influencing the treatment received, especially in medicine retailers, since this was found to be a major determinant of whether patients received an ACT. Thus, in developing interventions to improve malaria case management the results demonstrate the importance of addressing both demand and supply-side influences on malaria treatment.

## Competing interests

The authors declare that they have no competing interests.

## Authors' contributions

LM designed the survey, undertook the data analysis and drafted the paper with assistance from BC, VW, BU and OO. BC undertook sampling and provided advice on data analysis. OE and EN supervised the survey activities, with oversight from BU and OO. VW and OO provided guidance throughout the entire process. All authors read and approved the final manuscript.

## Funding

The research was supported by the ACT Consortium, which is funded through a grant from the Bill & Melinda Gates Foundation to the London School of Hygiene and Tropical Medicine.

## References

[B1] World Health Organization Guidelines for the Treatment of Malaria 2010 Second Geneva, Switzerland

[B2] Zurovac D Rowe AK Quality of treatment for febrile illness among children at outpatient facilities in sub-Saharan Africa Ann Trop Med Parasitol 2006 100 283 296 10.1179/136485906X105633 16762109

[B3] Zurovac D Ndhlovu M Sipilannyambe N Chanda P Hamer DH Simon JL Snow RW Paediatric malaria case-management with artemether-lumefantrine in Zambia: a repeat cross-sectional study Malar J 2007 6 31 10.1186/1475-2875-6-31 17367518 PMC1832199

[B4] Onwujekwe O Uzochukwu B Dike N Uguru N Nwobi E Shu E Malaria treatment perceptions, practices and influences on provider behaviour: comparing hospitals and non-hospitals in south-east Nigeria Malar J 2009 8 246 10.1186/1475-2875-8-246 19863803 PMC2775747

[B5] World Health Organization WHO briefing on malaria treatment guidelines and artemisinin monotherapies 2006 Geneva, Switzerland

[B6] Dondorp AM Nosten F Yi P Das D Tarning J Lwin KM Ariey F Hanpithakpong W Lee SJ Ringwald P Silamut K Imwong M Chotivanich K Lim P Herdman T An Yeung SS Singhasivanon P Day NPJ Lindegardh N Socheat D White NJ Artemisinin resistance in *Plasmodium falciparum *malaria NEJM 2009 361 455 467 10.1056/NEJMoa0808859 19641202 PMC3495232

[B7] Zurovac D Njogu J Akhwale W Hamer DH Snow RW Translation of artemether-lumefantrine treatment policy into paediatric clinical practice: an early experience from Kenya Trop Med Int Health 2008 13 99 107 10.1111/j.1365-3156.2007.01980.x 18291008 PMC2592474

[B8] Jimmy EO Achelonu E Orji S Antimalarials dispensing by patent medicine dealers in rural settlements in Nigeria Public Health 2000 114 282 285 10.1016/S0033-3506(00)00346-2 10962592

[B9] ACT Watch Outlet Survey Report (Baseline) Federal Republic of Nigeria 12/08 2008 Abuja, Nigeria

[B10] Federal Republic of Nigeria National Antimalarial Treatment Policy 2005 Abuja, Nigeria

[B11] Federal Republic of Nigeria National Antimalarial Treatment Policy 2005 Abuja, Nigeria

[B12] Federal Republic of Nigeria Training Manual for Management of Malaria in Nigeria 2005 Abuja, Nigeria; Federal Ministry of Health, Abuja, Nigeria

[B13] Meremikwu M Okomo U Nwachukwu C Oyo-Ita A Eke-Njoku J Okebe J Oyo-Ita E Garner P Antimalarial drug prescribing practice in private and public health facilities in south-east Nigeria: a descriptive study Malar J 2007 6 55 10.1186/1475-2875-6-55 17480216 PMC1867820

[B14] Okeke TA Okeibunor JC Rural-urban differences in health-seeking for the treatment of childhood malaria in south-east Nigeria Health Policy 2010 95 62 68 10.1016/j.healthpol.2009.11.005 20004038

[B15] Onwujekwe O Hanson K Uzochukwu B Ezeoke O Eze Soludo Dike N Geographic inequities in provision and utilization of malaria treatment services in southeast Nigeria: diagnosis, providers and drugs Health Policy 2010 94 144 149 10.1016/j.healthpol.2009.09.010 19836852

[B16] Uzochukwu BSC Chiegboka LO Enwereuzo C Nwosu U Okorafor D Onwujekwe O Uguru NP Sibeudu FT Ezeoke OP Examining appropriate diagnosis and treatment of malaria: availability and use of rapid diagnostic tests and artemisinin-based combination therapy in public and private health facilities in south east Nigeria BMC Public Health 2010 10 486 10.1186/1471-2458-10-486 20712876 PMC2931470

[B17] Uzochukwu BSC Onwujekwe OE Soludo E Nkoli E Uguru NP The District Health System in Enugu State, Nigeria: An analysis of policy development and implementation CREHS research report 2009 http://www.crehs.lshtm.ac.uk/publications.html

[B18] Okeke TA Uzochukwu BSC Okafor HU An in-depth study of patent medicine sellers' perspectives on malaria in a rural Nigerian community Malar J 2006 5 97 10.1186/1475-2875-5-97 17078875 PMC1635422

[B19] Onwujekwe O Ojukwu J Uzochuckwu B Dike N Ikeme A Shu E Where do people from different socio-economic groups receive diagnosis and treatment for presumptive malarial in south-eastern Nigeria? Ann Trop Med Parasitol 2005 99 473 481 10.1179/136485905X51283 16004706

[B20] Ordinioha B Inyenaporo C Experience with the use of community health extension workers in primary care, in a private rural health care institution in South-South Nigeria Annals of African Medicine 2010 9 240 245 10.4103/1596-3519.70964 20935425

[B21] Bennett S Woods T Liyanage WM Smith DL A simplified general method for cluster-sample surveys of health in developing countries World Health Statistics Quarterly 1991 44 98 106 1949887

[B22] World Health Organization Guidelines for the Treatment of Malaria 2006 Geneva, Switzerland

[B23] StataCorp Stata: Release 11. Statistical Software 2009 College Station TX: StataCorp L.P

[B24] Rao JNK Scott AJ The analysis of categorical data from complex sample surveys: chi-squared tests for goodness-of-fit and independence in two-way tables J Am Statistical Assoc 1981 76 221 230 10.2307/2287815

[B25] Yeung S White NJ How do patients use antimalarial drugs? A review of the evidence Trop Med Int Health 2005 10 121 138 10.1111/j.1365-3156.2004.01364.x 15679555

[B26] Juma E Zurovac D Changes in health workers' malaria diagnosis and treatment practices in Kenya Malar J 2010 10 1 10.1186/1475-2875-10-1 PMC3022768 21214892

[B27] Wasunna B Zurovac D Goodman CA Snow RW Why don't health workers prescribe ACT? A qualitative study of factors affecting the prescription of artemether-lumefantrine Malar J 2008 7 29 10.1186/1475-2875-7-29 18252000 PMC2266770

[B28] Zurovac D Tibenaderana JK Nankabirwa J Ssekitooleko J Njogu JN Rwakimara JB Meek S Talisuna A Snow RW Malaria case-management under artemether-lumefantrine treatment policy in Uganda Malar J 2008 7 181 10.1186/1475-2875-7-181 18803833 PMC2556699

[B29] Kangwana BB Njogu J Wasunna B Kedenge SV Memusi Goodman CA Zurovac D Snow RW Malaria drug shortages in Kenya: a major failure to provide effective treatment Am J Trop Med Hyg 2009 80 737 738 19407116 PMC2679204

[B30] Rowe AK Ponce de Leon G Mihigo J Carolina A Santeli FS Miller NP Van-Dunem P Quality of malaria case-management at outpatient health facilities in Angola Malar J 2009 8 275 10.1186/1475-2875-8-275 19954537 PMC2795764

[B31] Dike N Onwujekwe O Ojukwu J Ikeme A Uzochuckwu B Shu E Influence of education and knowledge on perceptions and practices to control malaria in southeast Nigeria Soc Sci Med 2006 63 103 106 10.1016/j.socscimed.2005.11.061 16448735

[B32] Wiseman V Scott A Conteh L Stevens W Milligan P Determinants of provider choice for malaria treatment: lessons from The Gambia Soc Sci Med 2008 67 487 496 10.1016/j.socscimed.2008.04.007 18538458

[B33] Filmer D Pritchett LH Estimating wealth effects without expenditure data or tears: an application to educational enrolments in States of India Demography 2001 38 115 132 11227840 10.1353/dem.2001.0003

